# Dietary Selenomethionine Reduce Mercury Tissue Levels and Modulate Methylmercury Induced Proteomic and Transcriptomic Alterations in Hippocampi of Adolescent BALB/c Mice

**DOI:** 10.3390/ijms232012242

**Published:** 2022-10-13

**Authors:** Ragnhild Marie Mellingen, Lene Secher Myrmel, Josef Daniel Rasinger, Kai Kristoffer Lie, Annette Bernhard, Lise Madsen, Ole Jakob Nøstbakken

**Affiliations:** 1 Institute of Marine Research, Nordnesgaten 50, 5005 Bergen, Norway; 2 Institute of Biomedicine, University of Bergen, 5007 Bergen, Norway; 3 Department of Biology, University of Copenhagen, 1165 København, Denmark

**Keywords:** Methylmercury, dietary interaction, selenomethionine, proteomics, RNA sequencing

## Abstract

Methylmercury (MeHg) is a well-known environmental contaminant, particularly harmful to the developing brain. The main human dietary exposure to MeHg occurs through seafood consumption. However, seafood also contains several nutrients, including selenium, which has been shown to interact with MeHg and potentially ameliorate its toxicity. The aim of this study was to investigate the combined effects of selenium (as selenomethionine; SeMet) and MeHg on mercury accumulation in tissues and the effects concomitant dietary exposure of these compounds exert on the hippocampal proteome and transcriptome in mice. Adolescent male BALB/c mice were exposed to SeMet and two different doses of MeHg through their diet for 11 weeks. Organs, including the brain, were sampled for mercury analyses. Hippocampi were collected and analyzed using proteomics and transcriptomics followed by multi-omics bioinformatics data analysis. The dietary presence of SeMet reduced the amount of mercury in several organs, including the brain. Proteomic and RNA-seq analyses showed that both protein and RNA expression patterns were inversely regulated in mice receiving SeMet together with MeHg compared to MeHg alone. Several pathways, proteins and RNA transcripts involved in conditions such as immune responses and inflammation, oxidative stress, cell plasticity and Alzheimer’s disease were affected inversely by SeMet and MeHg, indicating that SeMet can ameliorate several toxic effects of MeHg in mice.

## 1. Introduction 

Methylmercury (MeHg) is a wide-spread environmental contaminant, which bioaccumulates along the food-chains, especially in aquatic organisms [1,2]. MeHg exposure in humans occurs mainly through fish and seafood consumption. MeHg is absorbed in the intestines, distributed to all organs and excreted via feces [3,4]. A range of toxic effects by MeHg has been observed in rats and mice, such as locomotor and coordination impairments, increased oxidative stress and hippocampal neurodegeneration [5,6,7,8]. The developing brain is particularly vulnerable to MeHg toxicity [9]. In humans, dose-dependent effects related to degeneration of neurons, neurodevelopmental delay, ataxia, tremor, hearing loss and sensory deficit have been observed and connected to mechanisms like inhibited protein synthesis, oxidative stress, altered calcium homeostasis, impaired neurotransmitter function and microtubule disruption [10,11].

Nutrients present in seafood may interact with MeHg and affect its toxicity [12]. Selenium is an essential trace element derived from fish and seafood, which is incorporated in selenoproteins [13,14,15,16]. Selenium is involved in several important functions and processes in the body, such as the antioxidant defense, immune function and production of thyroid hormones [13,17]. Selenium, was hypothesized to protect against mercury toxicity already 50 years ago [18], but as many other nutrients, selenium is toxic when ingested at high doses. 

Inorganic mercury (Hg^2+^) has a very high affinity to selenium [19], leading to a strong sequestering also of MeHg by selenium and a specific inhibition of selenoenzymes by MeHg [20]. Proposed neurotoxic mechanisms of MeHg include inhibition of selenoenzymes causing oxidative stress, as well as sequestering of selenium in the brain inducing selenium deficiency and thereby promoting apoptosis and impaired re-synthesis of selenoproteins [20,21]. These mechanisms imply that replenishing selenium may, at least in part, reverse toxic effects of MeHg [22,23,24]. Molar excess of selenium in relation to mercury has therefore been proposed by several researchers as a protective measurement considering human risk of mercury toxicity when assessing seafood content [25,26]. The organic selenium compound, selenomethionine (SeMet), is an abundant selenium species in fish [27,28]. SeMet has previously been shown to reduce the accumulation of mercury in zebrafish and to counteract the MeHg mediated alterations of the brain proteome [29,30]. 

In the context of next generation risk-benefit assessments, toxicogenomic tools have been gaining importance [31]. Comprehensive bioinformatic anchoring of omics profiling data to regulatory networks, cellular pathways, and biological functions allows for an efficient biological interpretation of expression data and the prediction of mechanistic toxicological effects and modes of action, respectively [32,33]. In particular, the application of multi-omics relying on a cross-validation of proteomics findings with transcriptomics data (and vice versa) was found to be well-poised for the characterization and elucidation of mechanisms of action of neurotoxic xenobiotics in developing mice [34,35,36,37].

We hypothesize that addition of selenium to the diet of BALB/C mice will affect the general uptake of MeHg, and the molecular pathways in which MeHg exerts its effects in the brain. Therefore, by using a combination of measured tissue levels of mercury and next generation toxicogenomic tools, we investigated the combined effects of SeMet and MeHg on mercury accumulation in tissues and the effects co-exposure of these compounds exerts on the hippocampal proteome and transcriptome following dietary exposure in adolescent mice. The inbred strains of BALB/c mice were chosen for this experiment due to their sensitivity to MeHg and established position as model species for toxicological research [36,37,38,39,40]. 

## 2. Results

The present study investigated the combined effects of SeMet–MeHg co-exposure on mercury accumulation in tissues and aimed to elucidate the mechanisms by which MeHg and SeMet interacts in the hippocampal proteome and transcriptome in the developing brain of adolescent mice. We recorded and analyzed alterations of proteins and transcripts in hippocampi of adult mice, which through their adolescence were fed diets spiked with MeHg in doses of 0, 0.28 (LD) or 5 mg Hg kg^−1^ (HD) in the presence and absence of supplementary SeMet (2.5 mg Se kg^−1^, see Table 1 for experimental design). Main effects of MeHg from this experiment were described previously [41]. Briefly, we demonstrated that most MeHg-induced effects on protein abundance and transcript expression levels in hippocampus were dose-dependent. We also described that at the pathway level, functions involved in neurotoxicity, energy metabolism and oxidative stress were affected both at high and low dose MeHg exposures. In the present study, we focus on the SeMet–MeHg interaction effects only. 

### 2.1. Feed Intake and Body Weight

During the experiment, no mortality or illness was observed in any of the mice, regardless of diet fed. Feed intake, body weight development, organ weights, selenium levels and other physiological parameters were assessed during the trial (Figures S1 and S2 and Table S1). No differences were observed in feed intake between any of the dietary treatments. The total MeHg exposure was approximately 0.036 mg MeHg kg^−1^ bw^−1^ day^−1^ (MeHg LD), 0.033 mg MeHg kg^−1^ bw^−1^ day^−1^ (Se + MeHg LD) and 0.66 mg MeHg kg^−1^ bw^−1^ day^−1^ (MeHg HD and Se + MeHg HD). No significant differences were observed in body weight development, initial, or final body weight between the groups. Apart from a main effect of MeHg on kidney weights, with lower kidney weights (*p* = 0.03) in the mice of the MeHg HD and Se + MeHg HD groups (Table S1), no differences in organ weights were detected. 

### 2.2. Mercury Tissue Levels and Excretion

**Figure 1:**
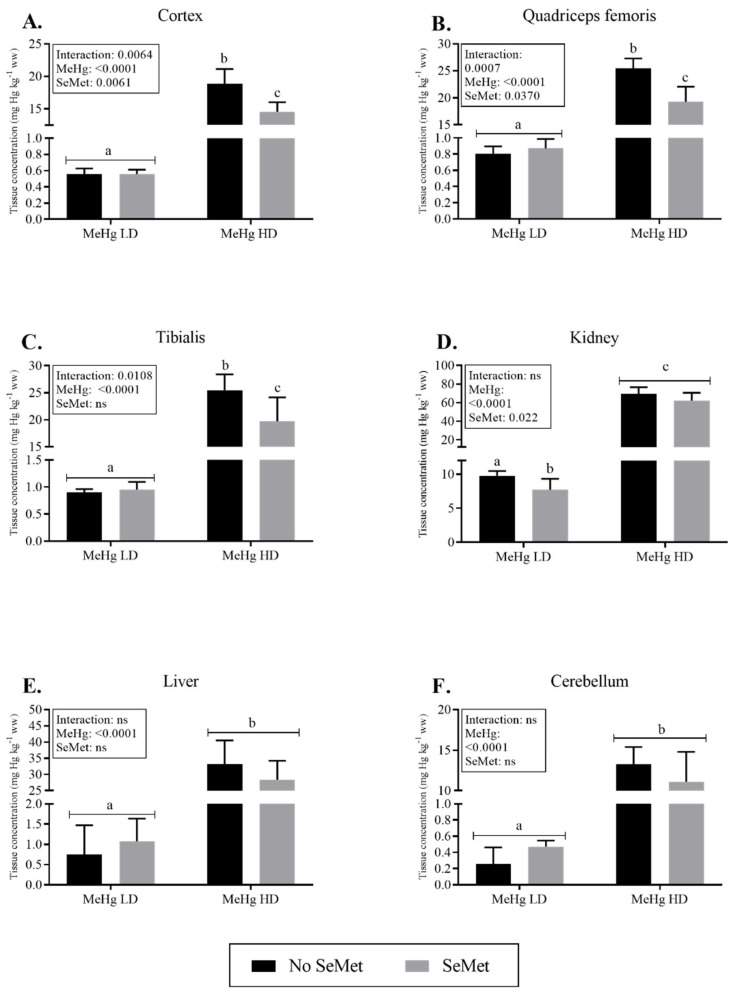
Total Hg concentration (mg Hg kg^−1^ ww) in cortex (**A**), quadriceps femoris (**B**), tibialis (**C**), kidney (**D**), liver (**E**) and cerebellum (**F**) of adolescent BALB/c mice exposed to dietary MeHg (0.28 and 5 mg Hg kg^−1^ feed) with or without supplementary SeMet for 11 weeks. All results are presented as means with 95% CI (*n* = 6). The box in each graph denotes *p*-values from two-way ANOVA analysis showing MeHg (main effect), SeMet (main effect) and interaction effect between MeHg and SeMet. Different lowercase letters represent statistical significance (*p* < 0.05) between the groups analyzed with the post hoc test Tukey’s multiple comparisons test. Statistics are based on log transformed (log_10_) data (**A**–**D**) and robust statistics (**E**,**F**) while figures present raw data. The bars representing mercury levels in cortex (**A**) have been presented previously for the groups MeHg LD and MeHg HD [41] but are still presented here for comparative purposes between the mercury levels in mice with and without the supplementary SeMet in the diet. Abbreviations: MeHg, methylmercury; SeMet, selenomethionine; LD, low dose; HD, high dose; ns, not significant.

Effects of SeMet–MeHg co-exposure on mercury tissue levels were assessed primarily in different sections of the brain (cortex and cerebellum), but also in other tissues known to accumulate mercury including the muscles tibialis and quadriceps femoris, the kidneys and the liver. The tissue levels of mercury increased dose-dependently in all organs observed. Simultaneous dietary intake of SeMet and MeHg significantly (*p* < 0.05) reduced the levels of mercury in cortex and muscles in the HD group (Figure 1). Mercury levels in the Se + MeHg HD group compared to the MeHg HD group were reduced by 22%, 23% and 24% in tibialis, cortex and quadriceps femoris, respectively. Additionally, reduced level of mercury by SeMet-exposure was observed in the Se + MeHg LD-group compared to the MeHg LD, but only in kidneys. 

**Figure 2:**
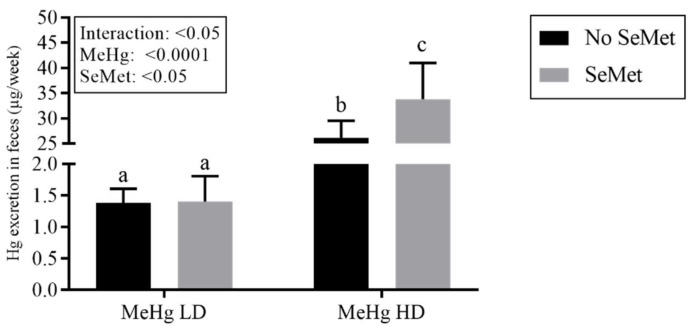
Total Hg concentration in feces after one-week continuous sampling (µg Hg/week). The results are presented as means with 95% CI (*n* = 6). These data did not pass the assumption of heterogeneity of variance and was therefore analyzed using robust ANOVA. The box denotes *p*-values from two-way ANOVA analysis showing MeHg (main effect), SeMet (main effect) and interaction between MeHg and SeMet. Different lowercase letters represent statistical significance (*p* < 0.05) between the groups analyzed with post hoc test (mcp2 in R). Abbreviations: MeHg, methylmercury; SeMet, selenomethionine; LD, low dose; HD, high dose; ns, not significant.

To assess mercury excretion, feces from all mice were collected during the last week of the experiment (week 11) and mercury levels were measured. The data did not meet the assumption of homogeneity of variance and were analyzed using robust two-way ANOVA. A borderline significant interaction was detected (*p* = 0.04), with an increased concentration of mercury measured in the feces of mice exposed to MeHg HD and SeMet concomitantly (Figure 2). 

### 2.3. Proteomics, Transcriptomics and Pathway Analyses

The effects of MeHg LD and HD has been described previously [41], thus present analyses primarily focus on the interaction effect between SeMet and MeHg HD. To elucidate molecular mechanisms underlying SeMet and MeHg interactions, we performed proteomic (*n* = 4/group) and RNA sequencing (*n* = 5/group) analyses of brain tissue of adolescent mice. All raw data of both transcriptomics and proteomics experiments were deposited in public repositories. These data were analyzed further using Qlucore Omics Explorer (QOE); analysis outputs are provided in Tables S2 and S3. Two-way ANOVA (Table 2) revealed an effect of MeHg HD on 128 proteins and 1775 transcripts (*p* < 0.05) and a SeMet main effect in 95 proteins and 1572 transcripts (*p* < 0.05). Interaction effects of SeMet and MeHg HD were observed for 149 proteins (*p* = 0.05) and 916 mRNA transcripts (*p* < 0.05) (for full list see Table S4 and S5). 

**Figure 3:**
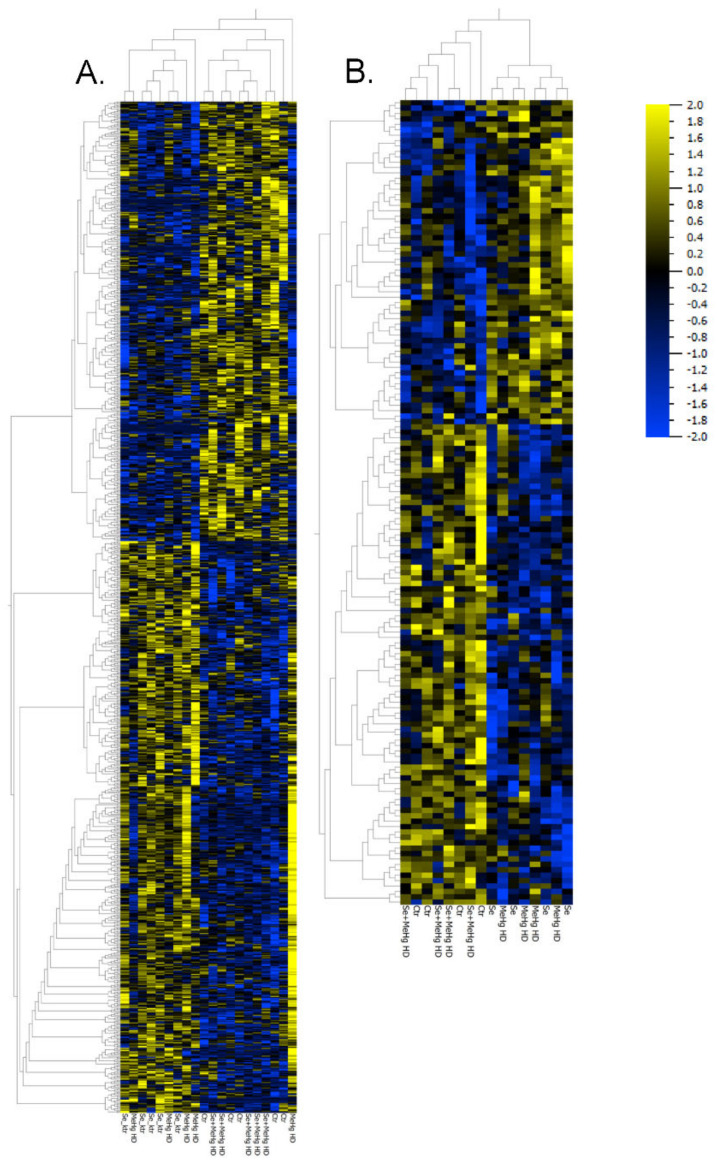
Heatmaps and hierarchical clustering analysis (HCA) displaying significant MeHg HD-SeMet interaction effect in 916 RNA transcripts (*p* < 0.05) (**A**) and in 149 proteins (*p* < 0.05) (**B**) (ANOVA, hierarchical clustering, Pearson correlation).

The proteins and RNA transcripts, for which interaction effects were noted (149 proteins/916 RNA transcripts) were organized in a heatmap and hierarchical clustering analysis was performed (Figure 3). The clustering analyses showed that the expression levels of RNA and proteins in hippocampus of the mice fed a diet supplemented with SeMet showed a similar expression pattern to mice fed only MeHg HD. Interestingly, the Se+MeHg HD group clustered with the Ctr group, indicating similar protein and RNA expression patterns in mice treated with SeMet and MeHg in combination and mice fed a control diet. This demonstrates that the combination of SeMet and MeHg HD attenuate the altered protein and transcript responses seen by SeMet or MeHg HD exposure alone.

Using the proteins and RNA transcripts showing significant interaction effects between SeMet and MeHg HD (149 proteins/916 RNA transcripts) as input, enrichment analyses were performed to further investigate potentially affected pathways, suggested diseases and functions and upstream regulators (Ingenuity Pathway Analysis software, IPA, Quiagen Bioinformatics, CA, USA). A summary of the top five most significant findings in each category (diseases and functions, canonical pathways and upstream regulators), with corresponding level of statistical significance and molecules from the dataset are presented in Table 3. The full list of pathways and upstream regulators can be found in Table S6–S9. 

We grouped the main findings of the IPA analysis into the following categories: (i) inflammation/immune response, (ii) cell plasticity, (iii) oxidative stress and (iv) Alzheimer’s disease. Furthermore, regulated proteins and RNA transcripts that showed significant interaction effects (between SeMet and MeHg HD) were grouped into the same respective categories. The specified categories were selected based on the IPA analysis, their involvement in well-known mechanisms of both MeHg and selenium previously described in the literature and to give examples on how SeMet and MeHg specifically can interact on protein and RNA expression. Examples of regulated features within these categories and corresponding *p*-values and direction of regulation are displayed in Table 4.

## 3. Discussion

In this study, we showed that concomitant exposure of dietary SeMet with MeHg, can reduce tissue levels of mercury in the brain and other target tissues in BALB/c mice. Using both proteomics and transcriptomics approaches, we further found that when dietary SeMet was administered in combination with MeHg protein, transcript responses induced by MeHg were restored to levels observed in the control group. These interactions were, through pathway analyses, further shown to indicate potential ameliorating effects of SeMet on MeHg-affected pathways such as inflammation and immune response, cell plasticity, oxidative stress and Alzheimer’s disease. 

Reduced accumulation of mercury has previously been shown to occur following selenium exposure in experiments using zebrafish [29], seabream [42], mice [43,44] and rats [45,46]. Due to the high affinity of MeHg to selenium [47], the Se-Hg complex will form in the intestine and subsequently reduce the absorption and increase fecal excretion of mercury. In the present study a statistically significant increase of mercury in feces of SeMet- and MeHg-exposed mice was observed for the high dose. This is in line with several previous studies which have reported increased excretion of mercury after selenium supplementation [29,48,49,50,51]. Moreover, selenium may contribute to MeHg demethylation in the intestines whereby uptake of MeHg in the body is reduced and the percentage of the less bioavailable inorganic mercury is increased [42,46,52]. Also, redistribution of mercury by selenium to less critical target organs has been a proposed mechanism of the protective Se-effect [53,54], e.g., redistribution to fur, a compartment prone to high mercury levels [55,56]. 

We further observed that dietary supplementation of SeMet can alleviate MeHg-induced proteomic and transcriptomic alterations in hippocampi of BALB/c mice, leading to expression patterns similar to mice fed the control diet (Figure 3). This effect was first described in a study by Rasinger, Lundebye, Penglase, Ellingsen and Amlund [30] where it was found that in brains of zebrafish exposed to 10 and 5 ug/g MeHg and SeMet, respectively, the dysregulation of proteins induced by MeHg was restored to control levels when MeHg exposure occurred in the presence of SeMet. 

Increased oxidative stress is a well-known molecular effect of MeHg, and selenium’s role as a redox regulator is also well known [21,57,58]. In this study, we showed interacting effects between SeMet and MeHg HD on features related to oxidative stress and antioxidant activity. The pathway of glutathione redox reactions was predicted by IPA to be affected in the proteomic analyses based on the involvement of the proteins glutathione peroxidase (GPX4), microsomal glutathione S-transferase 3 (MGST3) and peroxiredoxin-6 (PRDX6) from our dataset. Thioredoxin reductase 1 (TRXR1) was also among the significantly regulated proteins in our dataset with an interacting effect between SeMet and MeHg HD. Decreased Trxr activity and expression levels of Trxr1 have been shown on several occasions by MeHg-exposure [57,59,60], while exposure to selenite led to restored mRNA levels of Trxr1 [59]. Likewise, GPX activity and Gpx4 expression levels have been observed to decrease by MeHg exposure [60,61,62]. This shows that our findings on the reduced expression of essential selenoenzymes such as TRXR1 and GPX4 by MeHg exposure oxidative are in line with the literature. 

In our study, we found canonical pathways involving the complement system, inflammasome pathway and the pyroptosis signaling pathway, which suggests connections to inflammatory responses and immune system. MeHg HD upregulated expression of several genes related to the complement system, like complement C1q subcomponent subunit a-c (C1qa, C1qb and C1qc), and when SeMet was present alone or in combination with MeHg HD the expression was similar to levels in control-fed mice. C1qb was also regulated in the same manner on protein level. Additionally, interleukin 1β (IL-1β) was upregulated on RNA level by MeHg HD and not in SeMet or Se+MeHg HD, indicative of a MeHg-induced inflammatory process alleviated by SeMet. Mercury and MeHg exposure in humans have previously been related to induced inflammatory responses by proinflammatory cytokine expression [63]. Studies on selenium have in contrast revealed protecting mechanisms on inflammation and immune response such as reduced intensity of inflammatory markers like IL-6, IL-10 and TNF-α [64]. Low selenium levels have been connected to different inflammatory states and infections possibly indicating that stress, inflammation and infections may be influenced by selenium availability [65]. In addition, studies have reported that selenium may recover MeHg-induced immune suppression through diminishing the oxidative stress, cellular dysfunctions of B cells and reduced antioxidant levels caused by MeHg. Overall, this suggests that the production of oxidative stress is a mechanism of MeHg-immunotoxicity which could be counteracted by selenium [66]. According to our findings, and in line with previous research, we suggest that MeHg influence inflammation and immune responses by increasing the expression of IL-1β, possibly leading to an increased expression of components of the classical complement system (here C1q a-c). 

Pathways possibly connected to mechanisms related to cell plasticity, including remodeling of epithelial adherens junctions and semaphoring signaling in neurons were affected. Two different tubulin proteins were detected in the dataset of interaction effects between MeHg HD and SeMet; tubulin alpha-8 chain (TUBA8) and tubulin alpha-4A chain (TUBA4A), together with microtubule-associated proteins RP/EB family member 1 and 2 (MAPRE1 and MAPRE2) and two plexin proteins (plexin-A1;PLXA1 and plexin-B1;PLXB1). TUBA8 and TUBA4A are major constituents of microtubules while MAPRE1 and MAPRE 2 are involved in the regulation and dynamics of the microtubule cytoskeleton and in the mitotic spindle. PLXNA1 and PLXNB1 exerts functions related to semaphorin signaling, remodeling of the cytoskeleton and axon guidance (and thus cell growth and migration). Disturbances related to cell plasticity of neuronal cells like the degeneration of axons [67], inhibition of tubulin synthesis, reduced levels of microtubule-associated proteins and disruption, depolymerization and destabilization of microtubules [68,69,70,71], decreased cell adhesion and reduced branching of cells [72], are all known toxic effects of MeHg. The effects of mercury can thus lead to neurotransmitter dysfunction and blocking of the cytoskeletal assembly process [73]. As tubulin members have been observed to be downregulated by MeHg [74], dietary selenium given as SeMet resulted in an upregulation of these in the proteome of zebrafish [30]. Selenoproteins appear to be involved in the maintenance of microtubule stability through the direct interaction between Selenoprotein P and tubulin alpha 1a (TUBA1A) [75,76]. Our findings show that the proteins involved in cell plasticity were in most cases regulated by MeHg, but also in some cases by SeMet alone. However, all the proteins displayed expression levels similar to that of the control group in mice that received both MeHg and SeMet. This is a strong indication of a protective effect on proteomic alteration when the two elements are administered together and that the protective effect of selenium on MeHg-toxicity also concerns features related to cell plasticity, possibly related to microtubule function and axon guidance. 

Several upstream regulators which can be linked to Alzheimer’s disease (AD) were suggested through pathway analysis as possible driving forces to the changes observed in our dataset. On the protein level, the detected upstream regulators include: microtubule-associated protein tau (MAPT) (also found on RNA level), presenilin-1 (PSEN1), amyloid-beta precursor protein (APP), myelin regulatory factor (MYRF) and serine/threonine-protein kinase mTOR (also found in zebrafish exposed to MeHg and SeMet, respectively [30]). All detected regulators were shown to have associations to AD in the literature [77,78,79,80,81]. The suggested upstream regulators on RNA level: lysine-specific histone demethylase 1A (Kdm1a), alpha-N-acetylneuraminide alpha-2,8-sialyltransferase (St8sia1) and beta-1,4 N-acetylgalactosaminyltransferase 1 (B4galnt1) can also be linked to AD. These upstream regulators are involved in the pathogenesis and molecular alterations present in AD in different manners such as through the formation of amyloid/senile plaques (APP and PSEN1) and the development of neurofibrillary tangles (MAPT/Tau, PSEN1) [77,78,79]. mTOR signaling has been observed to increase in the brain of AD patients and in the hippocampus of transgenic mice with AD, while increased expression of MYRF mRNA has been detected at early and late stages of AD [80,81]. Furthermore, proteins and RNA transcripts significantly showing interaction effects in our dataset can be linked to AD, such as apolipoprotein E (APOE). APOE was significantly (*p* < 0.05) upregulated by MeHg HD, and expression levels normalized to control levels by SeMet alone or in combination with MeHg HD. APOE has been extensively linked to AD through its involvement in amyloid and plaque deposition in the brain, mitochondrial dysfunction and neurotoxicity, stimulation of MAPT/Tau phosphorylation and impairment of neuronal plasticity, especially in the form of the APOE ε4 isoform [82]. Further, glial fibrillary acidic protein (GFAP) is also associated with brain Aβ pathology as a potential early marker of AD pathogenesis in plasma [83], expression of GFAP in hippocampus is associated with AD pathogenesis [84] and increased transcription levels of GFAP is associated with AD progression [85]. GFAP showed significant interaction effect in both proteins and RNA transcripts in our dataset. MeHg HD significantly upregulated the expression of GFAP as a protein and RNA transcript, while SeMet normalized the expression to that of control levels both alone and in combination with MeHg HD.

Both selenium and mercury have been linked to the development and pathogenesis of AD, however, the clinical relevance and connections have been conflicting [76,86,87,88,89,90,91]. The many similarities between mercury toxicity and AD pathology have recently been emphasized by Siblerud et al. [92] and Bjørklund et al. [86]. Resemblances between the disease and mercury toxicity through immune response and inflammatory processes such as complement activation, cytokine expression and increased GFAP and IL-1 have been noted [92]. Also, links between AD and mercury toxicity regarding antioxidant system, oxidative stress and microtubule structure, assembly and consequently neuron degeneration were found [86], all corresponding to findings in our study, even concluding that mercury itself can be the cause of AD [92]. On the other hand, different protective mechanisms of selenium on AD have been investigated related to the reduction of amyloid β production and toxicity, antioxidative defense against ROS which are associated with progression of AD and reduced levels of MAPT/tau and phosphorylated MAPT/tau to mention some [76,90,93]. However, testing of selenium as a therapeutic agent in the prevention of dementia in elderly men has shown no effect [94]. Previously, the upstream regulators APP, MAPT/Tau and PSEN1 have been found to be inversely regulated by MeHg and SeMet in the brain of zebrafish [30], as seen also in our trial. Increased seafood consumption has been negatively correlated with AD among APOE ε4 carriers, despite higher levels of mercury in the brain. Increased mercury levels from seafood were positively correlated with elevated selenium levels, further strengthening the hypothesis of a protective effect from selenium on mercury AD-like pathogenesis [95].

SeMet seems to have a protective effect on MeHg-induced regulations of APOE and GFAP (on protein and RNA level) and interacting effects on a range of upstream regulators related to AD, in addition to several features already mentioned involved in immune response/inflammation, redox balance and cell plasticity which demonstrates possible associations between selenium, MeHg and AD in the present study. The hippocampus, the brain section of interest in this study, is also one of the first regions of the brain damaged in AD [96]. However, our findings do not necessarily implicate that the mice developed AD, but it may indicate that they developed Alzheimer-like molecular responses in hippocampus.

### Limitations

It must be taken into consideration that the number of individuals in each group included in the omics analyses is limited. Furthermore, the use of unadjusted *p*-values instead of *p*-values adjusted for multiple testing (*q*-values) were chosen as this has been proposed to increase the sensitivity of omics analyses [97]. However, using unadjusted *p*-values may lead to a higher rate of false positive findings which should be taken into consideration when interpreting the results. In general, we cannot confirm the cause of a certain regulation in protein or RNA expression and whether it is the direct interaction of MeHg and SeMet on the specific features or if it is a compensatory action in response to other molecular mechanisms caused by the two elements in the brain of the mice. Nor can we rule out that the reduced levels of mercury in tissues in response to increased SeMet in the diet may be the cause for changes in gene expression. However, the combined use of proteomic and transcriptomic tools strengthens the relevance of the findings.

## 4. Material and Methods

The present study reports in parts on data obtained from a previous feeding trial with male BALB/c [41]. In the sections below, a brief account of material and methods published earlier is given; material, methods and analyses not previously reported on are provided in full. 

### 4.1. Experimental Design and Sampling

As previously described [41], male BALB/c mice (Taconic Biosciences, Ejby, Denmark) arrived at the laboratory at the age of 2–3 weeks weighing 10.3 ± 1.4 g. The experiment and animal facility were approved by the Norwegian Food Safety Authority (Mattilsynet; FOTS ID:12400). Following five days of acclimation, the mice were divided in six treatment groups (*n* = 6 per group) based on body weight and fed experimental diets three times a week for 11 weeks. 

Experimental diets were based on the AIN-93G purified diet (Harlan Laboratories Ltd., Indianapolis, IN, USA). Using a two-by-three factorial design, mice were fed control diets or diets spiked with seleno-L-methionine (SeMet), MeHg or a combination thereof. The diets with SeMet supplementation are hereafter denoted with the prefix Se. The three levels of MeHg used are hereafter referred to as Ctr (no MeHg), LD (low dose) MeHg or HD (high dose) MeHg. Detailed information of Ctr, LD MeHg and HD MeHg diet is provided in [41] and Table 1 provides a summary of experimental design and supplemented levels of MeHg and SeMet. Desired concentrations of MeHg in the diets were 0.28 mg Hg kg^−1^ (LD) and 5 mg Hg kg^−1^ (HD). The chosen LD was based on low exposure doses from previous trials where effects of MeHg have been detected [98,99] and the HD was chosen to be certain of MeHg effect without mortality or severe illness in the mice, based on experience from previous mice trials at our institute (data not published). A MeHg HD stock was made with a molar ratio of 1:1 MeHg (Methylmercury(II)chloride, Sigma-Aldrich, Darmstadt, Germany) and cysteine (L-cysteine from non-animal source, Sigma-Aldrich, Darmstadt, Germany). The stock was further diluted to prepare the LD stock, and a cysteine stock was prepared for the Ctr and Se diets. For further details about the preparation of diets see [41]. Supplementary selenium was added to the feed in the form of seleno-L-methionine (Sigma-Aldrich, Darmstadt, Germany). The concentration of SeMet in Se, Se+MeHg LD and Se+MeHg HD was adjusted to 2.5 mg Se kg^−1^, which corresponds to a molar ratio of 1.26:1 of selenium and mercury, respectively, when compared to the HD level of Hg. This molar ratio was chosen because a molar excess of selenium in relation to mercury is considered beneficial [25] and the ratio corresponds to the one used in a previous trial where ameliorating effects of selenium on MeHg toxicity were seen [29].

Inductively coupled plasma mass-spectrometry (ICP-MS) was used as described elsewhere [100] to verify selenium and mercury levels in diets. The selenium levels in control feeds were determined to be 0.088 mg kg^−1^ (*n* = 9), mercury levels in the control feeds were below the limit of quantification (LOQ) for the instrument (*n* = 4). The LD-, HD-MeHg and selenium levels in diets were in accordance with nominal concentrations within the uncertainty range (±20%) of the method (*n* = 4). 

Bilateral thoracotomy and cardiac puncture after sedation was the chosen method for sacrifice. The organs deemed most relevant for mercury toxicity and accumulation were collected. Liver, kidneys, hippocampus, cortex, cerebellum, tibialis and quadriceps femoris were sampled, weighed, snap-frozen in liquid nitrogen and stored at −80 °C until further processing. Feces from the 10th week of the experiment were collected from the bedding material of each cage, homogenized, and then stored at −20 °C for later analyses.

### 4.2. Mercury and Selenium Determination

Total mercury (THg) was analyzed in kidneys, muscles, cortex and feces by direct mercury analysis (DMA-80, Milestone, Sorisole, Italy) as described elsewhere [41,101]. THg and selenium levels in diets, liver and cerebellum were determined by inductively coupled plasma mass spectrometry (ICP-MS; Thermo iCAP Q, ThermoFisher Scientific, Waltham, MA, USA) as described by Julshamn et al. [100]. The LOQ of this method is 0.005 mg kg^−1^ for mercury and 0.01 mg kg^−1^ for selenium. Total MeHg exposure through the diet was calculated using the following formula:

$$\frac{\mathrm{MeHg}\;\mathrm{concentration}\;\times \;\mathrm{average}\;\mathrm{total}\;\mathrm{feed}\;\mathrm{intake}\;\mathrm{in}\;\mathrm{each}\;\mathrm{group}}{\mathrm{Total}\;\mathrm{length}\;\mathrm{of}\;\mathrm{experiment}\;\left(77\;\mathrm{days}\right)\times \;\mathrm{average}\;\mathrm{final}\;\mathrm{body}\;\mathrm{weight}\;\mathrm{in}\;\mathrm{each}\;\mathrm{group}}$$

### 4.3. Proteomics Analysis and RNA Sequencing

Multi-omics analyses were done on hippocampus due to its sensitivity to MeHg toxicity [102,103]. A total of 24 hippocampus samples (four mice per exposure group) were prepared for proteomic analysis. Sample preparation and protein mass spectrometry were performed as previously described [104] following standard protocols and procedures at the Proteomics Unit at the University of Bergen, Norway (PROBE). The preparation of peptides, the equipment used, software, settings and false discovery rates have formerly been specified [41]. Detailed protein expression data are provided in Table S2. 

Five hippocampus samples from each group were included for RNA-sequencing (RNA-seq), harvested from the same mice as the proteomics samples. Procedures, kits, equipment, RNA integrity number, software and library specifications have previously been described [41]. All RNA transcripts can be found in Table S3. Raw RNAseq reads in addition to normalized read counts were submitted to the gene expression omnibus https://www.ncbi.nlm.nih.gov/geo (accession number GSE135381). 

### 4.4. Statistics and Bioinformatics

GraphPad Prism^®^ 7 (GraphPad Software Inc., La Jolla, CA, USA) was used to statistically evaluate tissue levels of selenium and Hg. All the accumulation data were log transformed (log_10_) and tested for homogeneity of variances using Spearman‘s test for heteroscedasticity. To detect significant differences between groups, two-way analysis of variance (ANOVA) followed by Tukey’s multiple comparisons post hoc test were performed. *p* < 0.05 was chosen as threshold for accepting statistical significance. The Ctr and Se groups were not spiked with MeHg and accordingly showed levels below LOQ after analyses. Therefore, only LD and HD with and without selenium are shown and assessed statistically in this study due to no variation in the Ctr and Se groups. Thus, the design for accumulation data was reduced to a two-by-two factorial design. Statistics based on the log transformed data are presented in the figures and text (Figure 1 and Figure 2), while the data are shown as not transformed. Data, which despite transformations did not meet assumptions for parametric tests, were subjected to robust statistical methods using the WRS2 package [105] in R vers. 3.5.3 [106] running in RStudio vers. 1.2.1335 [107]. 

Proteomic and transcriptomic data were statistically assessed in Qlucore Omics Explorer 3.5 (Qlucore AB, Lund, Sweden). Data were analyzed using two-way ANOVA comparing four groups. Unadjusted *p*-values of 0.05 were chosen for accepting statistical significance to increase the sensitivity of the omics analyses. However, this may lead to a higher rate of false positives which should be taken into consideration when interpreting the results. Hierarchical clustering analysis was further used to assess the expression patterns of the data. 

The software Ingenuity Pathway Analysis (IPA, Quiagen, Redwood City, CA, USA) was used to perform biological network analyses. *p* < 0.05 was chosen for accepting statistical significance, “humans, rats and mice” were the species of selection, and the settings for specification of tissues and cells were narrowed down to “nervous system”, “CNS cell lines” and “neuroblastoma cells”. “Core analysis” (using default settings) was performed on proteins and mRNA transcripts separately in each group, for further manual inspection and comparison. 

## 5. Conclusions

In our study, selenium in the form of SeMet reduced the levels of mercury in several tissues and increased excretion in feces upon dietary MeHg exposure, indicating an inhibitive effect of SeMet on the absorption or metabolism of mercury. Further, proteomic and transcriptomic results revealed counteracting effects of SeMet and MeHg on protein and RNA expression patterns. A range of pathways and molecular targets was oppositely influenced by MeHg and SeMet, of which some could be related to immune response and inflammation, cell plasticity, oxidative stress and Alzheimer disease. Based on findings reported in literature and the data obtained in the present study, it can be hypothesized that selenium when present in diets contaminated with MeHg exerts a protective effect on MeHg-induced alterations of the brain proteome and transcriptome. 

Although MeHg affects neurodevelopment detrimentally, most risk–benefit assessments recommend intake of seafood, which may contain high levels of MeHg. Data from this study, in addition to other studies provide evidence to suggest that the reversal of MeHg toxicity by the marine nutrient selenium should be taken into account when seafood is risk assessed [108], especially when assessing fish species which have a high mercury and selenium content.

## Figures and Tables

**Table 1:** Experimental design and dosages of MeHg and SeMet.

	No SeMet	SeMet ^3^
No MeHg	Ctr (*n* = 6)	Se (*n* = 6)
Low dose (LD) ^1^	MeHg LD (*n* = 6)	Se + MeHg LD (*n* = 6)
High dose (HD) ^2^	MeHg HD (*n* = 6)	Se + MeHg HD (*n* = 6)

^1^ 0.28 mg Hg kg^−1^. ^2^ 5 mg Hg kg^−1^. ^3^ 2.5 mg SeMet kg^−1^. Abbreviations: Ctr, control; Se, selenium; SeMet, selenomethionine; MeHg, methylmercury; LD, low dose; HD, high dose.

**Table 2:** Summary of the numbers of differentially expressed proteins and RNA transcripts (*p* ≤ 0.05) according to different statistical comparisons in Qlucore Omics Explorer. The proteins and transcripts indicated by numbers in bold are used for further analysis in the Ingenuity Pathway Analysis software (IPA), upon which findings are described in Table 3.

Two Group Comparison			Proteomics	RNA Sequencing
Group	Vs.	Group	Proteins (*n*)	Transcripts (*n*)
			*p* = 0.05	*p* = 0.05
Ctr		MeHg HD	120	1411
Ctr		Se	79	856
Ctr		Se+MeHg HD	137	1043
Se		MeHg HD	66	1927
Se		Se+MeHg HD	121	938
HD		Se+MeHg HD	113	1349
Multi Group Comparison (ANOVA)			Proteomics	RNA sequencing
Effect		Groups included	Proteins (*n*)	Transcripts (*n*)
			*p* = 0.05	*p* = 0.05
MeHg HD main effect		Ctr, MeHg HD, Se, Se+MeHg HD	128	1775
SeMet main effect		Ctr, MeHg HD, Se, Se+MeHg HD	95	1572
Interaction effect HD		Ctr, MeHg HD, Se, Se+MeHg HD	**149**	**916**

Abbreviations: ANOVA, analysis of variance; MeHg, methylmercury; SeMet, selenomethionine; Ctr, control; HD, high dose MeHg; Se, SeMet and no MeHg; SeHD, SeMet and high dose MeHg.

**Table 3:** Summary of diseases/functions, canonical pathways and upstream regulators from Ingenuity Pathway Analysis (IPA) based on the proteins (149 proteins) and RNA transcripts (916 RNA) showing significant (*p* < 0.05) interaction effects between SeMet and MeHg HD, listed according to *p*-values calculated by the software. Features in bold represent examples of proteins and RNA transcripts involved in cell plasticity, oxidative stress, immune response and Alzheimer’s disease further described in Table 4. ** *p*-value < 0.01, *** *p*-value < 0.001.

Proteomics			Transcriptomics		
*Diseases or functions annotation*	*p*-value	Molecules from dataset	*Diseases or functions annotation*	*p*-value	Molecules from dataset
Neuromuscular disease	***	ACP1, ATP2B2, CTNNA2, HSBP1, MBP, NRCAM, AP1S1, ATP5MG, FABP7, HSPA5, MOG, TPT1, **APOE**, ATP6V1A, GAP43, IDH3A, NDUFB5, VAMP1, ATP2B1, COX5A, **GFAP**, KCNAB2, NDUFS6	Quantity of neuroglia	***	Csf1r, Galns, Mmp12, Ptprc, Trem2, Cxcl2, Hdac2, Myd88, Sox2, Tyrobp, Daam2, Il1b, Neil3, Sox9, Fgf2, Kcnj10, Notch1, St8sia4
Progressive neurological disorder	***	**APOE**, GAP43, MBP, **PRDX6**, WDR7, CST3, **GFAP**, MOBP, SLC2A1, CTNNA2, HSPA5, MOG, SORL1, FAAH, MAG, NRCAM, TPT1	Quantity of leukocytes	***	Alcam, Csf1r, Neil3, **C1qa**, Il1b, Pycard, C4a/C4b, Il27ra, Trem2, Cnr2, Myd88, Tyrobp
Disorder of the basal ganglia	***	ACP1, ATP2B2, CTNNA2, GRIN2B, MBP, TPT1, AP1S1, ATP5MG, FABP7, HSBP1, MOG, VAMP1, **APOE**, ATP6V1A, GAP43, IDH3A, NDUFB5, ATP2B1, COX5A, **GFAP**, KCNAB2, NDUFS6	Recruitment of phagocytes	***	Cxcl2, Il1b, Myd88, Tlr2
Morphology of the nervous system	***	ADAM22, BRSK2, CST3, GAP43, KIF5C, MOG, PTK2, SLC44A2, VAMP1, **APOE**, CHMP4B, CTNNA2, **GFAP**, MAG, NFASC, PTPRS, SORL1, ASPA, CNTNAP2, DHRS7B, GRIN2B, MAOA, NRCAM, RHEB, UBQLN2, ATP2B2, CSNK2A1, FKBP8, HSPA5, MBP, PRMT8, SLC2A1, UCHL1	Loss of neuronal progenitor cells	***	Foxo3, Neil3, Notch1
Alzheimer disease	***	**APOE**, GAP43, MOG, WDR7, CST3, **GFAP**, **PRDX6**, CTNNA2, MAG, SLC2A1, FAAH, MOBP, SORL1	Damage of central nervous system	***	Cnr2, Gpr34, Lepr, Sparc, Cx3cr1, **Il1b**, Mt3, Thbs1, Entpd1, Kcnk2, Olfml3, Tlr2, Fgf2, Lancl1, Pycard
*Canonical pathways*			*Canonical pathways*		
Remodeling of Epithelial Adherens Junctions	***	CTNNA2, EXOC2, **MAPRE1**, **MAPRE2**, **TUBA8**, **TUBA4A**	Complement System	***	**C1qa**, **C1qb**, **C1qc**, C3ar1, C4a/C4b, Itgam, Itgax
Phagosome Maturation	***	ATP6AP1, ATP6V1A, ATP6V1F, CALR, **PRDX6**, **TUBA8**, **TUBA4A**	Inflammasome Pathway	***	**Il1b**, Myd88, Naip, P2rx7, Pycard
Glutathione Redox Reactions	**	**GPX4**, **MGST3**, **PRDX6**	Pyroptosis Signaling Pathway	**	Casp4, Foxo3, Gsdmd, **Il1b**, Naip, Nol3, P2rx7, Pycard, Tlr2
Semaphorin Signaling in Neurons	**	FNBP1, **PLXNA1**, **PLXNB1**, PTK2	Urea Cycle	**	Ass1, Cps1
LPS/IL-1 Mediated Inhibition of RXR Function	**	ACSL1, ALDH18A1, **APOE**, FABP3, FABP7, MAOA, **MGST3**	Phospholipases	**	Gpld1, Hmox1, Plaat1, Plb1, Plcz1, **Prdx6**
*Upstream regulators*			*Upstream regulators*		
MAPT	***	UCHL1, MBP, ATP6V1A, **TUBA8**, NDUFS6, TPT1, HSPH1, GRIN2B, GAP43, **MAPRE2**, **PRDX6**, MOG, **GFAP**, SEC31A, HSPA5, **CIQB**, **TUBA4A**, WDR7	KDM1A	***	Tlr2, Tent5c, Slc43a3, Phf11, Il27ra, Apobec1, C4a/C4b, Trim21, C3ar1, Sox2, Cd22, Hmox1, Clec7a, Ctss, LCp1, Dock2, Itgax, Ptprc, Ccl4, Cst7, **Gfap**, Il21r, Ccl3l3, **C1qb**, Tyrobp, Glycam1
PSEN1	***	UCHL1, MBP, ATP6V1A, ACSL1, **TUBA8**, NDUFS6, TPT1, **APOE**, GRIN2B, **PRDX6**, **GFAP**, HSPA5, KCNAB2, **C1QB**, **TUBA4A**	MAPT	***	**Prdx6**, Tubb2a, Tlr2, Tent5c, Mt3, Slc43a3, Phf11, Abcg1, Ctsz, Tubb2b, Apobec1, C4a/C4b, Trim21, Map6, Thbs1, Pea15, C3ar1, Dbi, Camk2g, Cd63, CD22, Hmox1, Ctsd, Clec7a, Ctss, Lcp1, Dock2, Gucy1b1, Itgax, Ptprc, Ccl4, Cst7, **Gfap**, Il21r, Ccl3l3, **Il1b**, **C1qb**, Tyrobp, Gad1, Glycam1
mTOR	***	MBP, SERPINB1, ATP2B1, GAP43, MAG, UBE2O, MOG	ST8SIA1	***	C4a/C4b, C3ar1, Sox2, **C1qc**, **C1qa**, **Il1b**, **C1qb**
APP	***	UCHL1, MBP, ATP6V1A, **TUBA8**, NDUFS6, TPT1, **APOE**, GRIN2B, FABP3, GAP43, **PRDX6**, **GFAP**, HSPA5, **TUBA4A**	B4GALNT1	***	C4a/C4b, C3ar1, **C1qc**, **C1qa**, **Il1b**, **C1qb**
MYRF	***	MBP, MAG, MOG	L2HGDH	***	Clec7a, Itgax, Ccl4, Cd68, Ccl3l3

**Table 4:** Overview of selected features related to the overall categories of cell plasticity, oxidative stress, immune response and Alzheimer’s disease.

	Selected Features (abb.)	Level of Regulation	*p*-Value Interaction Effect	MeHg HD	Se	Se + MeHg HD
*Cell plasticity*	TUBA8	Protein	*			
	TUBA4A	Protein	**	*	*	
	MAPRE1	Protein	**			
	MAPRE2	Protein	*			
	PLXNA1	Protein	*	*		
	PLXNB1	Protein	**			
*Oxidative stress*	GPX4	Protein	*			
	PRDX6	Protein	*	*		
	Prdx6	RNA	*			
	MGST3	Protein	*		*	
	TRXR1	Protein	***	***		
*Immune response*	Il1b	RNA	**	**		
	C1qa	RNA	**	***		
	C1QB	Protein	**	**		
	C1qb	RNA	**	***		
	C1qc	RNA	**	**		
*Alzheimer’s disease*	APOE	Protein	*	*		
	GFAP	Protein	*	***		
	Gfap	RNA	**	***		

* *p*-value < 0.05, ** *p*-value < 0.01, *** *p*-value < 0.001. *p*-value from Two-way ANOVA interaction effect and post hoc test Tukey’s multiple comparisons test comparing all groups. Statistics are performed on log_10_ transformed data. The heatmap are displaying protein and RNA regulation (up = blue, down = red) and is calculated based on mean expression values in each group divided by the expression value of the Ctr group, thus showing the individual groups regulation in relation to control levels. Abbreviations: HD, high dose; Se, selenium; MeHg, methylmercury.

## Data Availability

Raw RNAseq reads in addition to normalized read counts were submitted to gene expression omnibus (accession number GSE135381). Detailed protein expression data will be made available upon request to the authors.

## Supplementary Materials

The following supporting information can be downloaded at: https://www.mdpi.com/article/10.3390/ijms232012242/s1.
